# Efficient production of human interferon beta in the white of eggs from ovalbumin gene–targeted hens

**DOI:** 10.1038/s41598-018-28438-2

**Published:** 2018-07-05

**Authors:** Isao Oishi, Kyoko Yoshii, Daichi Miyahara, Takahiro Tagami

**Affiliations:** 1 Biomedical Research Institute, National Institute of Advanced Industrial Science and Technology, 1-8-31, Midorioka, Ikeda, Osaka, 563-8577 Japan; 2 Animal Breeding and Reproduction Research Division, National Agriculture and Food Research Organization, Institute of Livestock and Grassland Science, 2 Ikenodai, Tsukuba, Ibaraki, 305-0901 Japan

## Abstract

Transgenic chickens could potentially serve as bioreactors for commercial production of recombinant proteins in egg white. Many transgenic chickens have been generated by randomly integrating viral vectors into their genomes, but transgene expression has proved insufficient and/or limited to the initial cohort. Herein, we demonstrate the feasibility of integrating *human interferon beta* (*hIFN-*β) into the chicken *ovalbumin* locus and producing hIFN-β in egg white. We knocked in *hIFN-*β into primordial germ cells using a CRISPR/Cas9 protocol and then generated germline chimeric roosters by cell transplantation into recipient embryos. Two generation-zero founder roosters produced *hIFN-*β knock-in offspring, and all knock-in female offspring produced abundant egg-white hIFN-β (~3.5 mg/ml). Although female offspring of the first generation were sterile, their male counterparts were fertile and produced a second generation of knock-in hens, for which egg-white hIFN-β production was comparable with that of the first generation. The hIFN-β bioactivity represented only ~5% of total egg-white hIFN-β, but unfolding and refolding of hIFN-β in the egg white fully recovered the bioactivity. These results suggest that transgene insertion at the chicken *ovalbumin* locus can result in abundant and stable expression of an exogenous protein deposited into egg white and should be amenable to industrial applications.

## Introduction

In recent decades, recombinant proteins have found use in industrial, agricultural, medical, and scientific applications. For such applications, cultured cells have mainly been used as hosts for production by ectopic expression of foreign genes. However, complex and expensive mechanical bioreactors are required for this type of mass production of recombinant proteins^1^. Therefore, cost-effective methods must be developed to produce large amounts of recombinant proteins. Plants and livestock animals have also been targeted as potential bioreactors owing to their potential advantages of low-cost and high-volume output^2,3^. Recombinant proteins, e.g., human blood factors, enzymes, and cytokines, have been generated in the organs of transgenic animals and plants, e.g., milk, blood, seeds, and roots^4–6^. Furthermore, certain of these protein products have been purified and commercialized for therapeutic use^7^.

Chickens may prove to be a useful livestock bioreactor if they can be made to abundantly produce specific recombinant proteins in their eggs because egg production is low in cost, and such proteins could be inexpensively obtainable in large yield^3,8–11^. In addition, the use of chickens as bioreactors would be expected to yield recombinant proteins having human-like glycosylation, i.e., with *N*-acetylneuraminic acid, although protein glycosylation in chickens differs somewhat from that in mammalian cell lines, which at present constitute the most common means of producing therapeutic recombinant proteins^12–14^. To date, a number of transgenic chicken lines that produce a recombinant protein in egg white have been established by embryonic microinjection of a retroviral or lentiviral vector. For example, human interferon-α2^15^, interferon-β^16^, erythropoietin^17^, epidermal growth factor^18^, lysozyme^19^, defensin^20^, and a humanized single-chain Fv-Fc antibody (ScFv-Fc)^16,21^ have been expressed and deposited in the egg white of transgenic hens. These studies suggest the possibility of using transgenic chickens as bioreactor systems; in most cases, however, the amount of foreign protein produced in egg white ranges from <0.1 to 0.2 mg/ml (reviewed in^11^). One possible explanation for the observed low-level transgene expression is the host silencing response to viral sequences—especially when a retrovirus is used as the vector^22^. Only the amount of ScFv-Fc in the egg white of the generation-zero (G0) transgenic hens could be considered to be large (4.0–5.6 mg/ml). (These hens were produced by injecting a large titer of a retroviral vector carrying the ScFv-Fc gene into the blood of embryos^21^.) However, this large-expression phenotype was not inherited or was suppressed in the eggs of subsequent generations (G1, G2, and G3). Furthermore, for transgenic hen lines produced to date by viral infection, the amount of foreign protein produced in the egg white of different individual G1 hens has varied widely, possibly owing to position effects resulting from transgene integration at random sites within the chromosomal DNA^11,23,24^. Moreover, detrimental effects on the development and health of these transgenic chickens may have occurred because of unfavorable ectopic gene expression and/or insertional mutagenesis that could have inactivated host genes^21,25,26^. Consequently, insufficient, unstable, and variable production of recombinant proteins in egg white of transgenic chickens—and the possible accompanying detrimental effects on the chickens themselves—need to be resolved before transgenic chickens can be considered as stable and practical industrial bioreactors that produce recombinant proteins. For this purpose, a finely tuned, generalized method that facilitates stable expression of an ectopic gene in a chicken bioreactor is required; to date, however, it has been difficult to do so with viral vectors as noted above. In addition, the inherent limitation on transgene size in viral transgenesis prevents the improvement of ectopic gene expression.

Conversely, although non-viral vectors have low transfection efficiencies compared with viral vectors, they are more flexible carriers because they do not have a strict size limitation for an ectopic gene and because they allow targeting of a specific chromosomal site. Non-viral vectors have been used to generate transgenic chickens with germline-competent cultured primordial germ cells (PGCs)^27–29^. Although transgene integration sites in the chicken genome were not strictly controlled in the aforementioned studies, Schusser and colleagues were able to use targeted PGCs, established by classical homologous recombination, and generated immunoglobulin heavy chain targeted chickens^30^. Recent exploitation of genome-editing technology has drastically increased the efficiency of gene targeting in PGCs, allowing for increased germline transmission efficiency and for efficiently generating transgenic chickens^31,32^. Because site-specific transgene integration can avoid negative position effects, valuable recombinant proteins should be able to be abundantly and stably produced in chicken eggs^33,34^. Insertion of exogenous DNA into a major oviduct-specific gene would be the preferred method because it enables efficient transgene expression as well as the deposition of the recombinant protein specifically in egg white. In addition, this type of procedure should be less detrimental to the health of the transgenic chicken compared with ubiquitous expression of the recombinant protein in the animal^35–37^. In this respect, the oviduct-specific ovalbumin gene (*OVA*), which encodes the most abundant protein in egg white (~50% of the total egg-white protein), is the preferable host gene for insertion of a foreign gene; notably, however, prior to this report neither *OVA* targeting nor specific targeting of a chicken gene to generate a chicken bioreactor had been realized.

In this study, we produced human interferon-β (hIFN-β) in egg white by integrating *hIFN-*β at the translation initiation site of the *OVA* locus. We first introduced *hIFN-*β cDNA into PGCs via CRISPR/Cas9-mediated genome editing and then generated knock-in (KI) hens as progeny of the germline G0 chimera roosters that had been transplanted with the KI PGCs at the embryonic stage. The KI hens expressed hIFN-β in their tubular glands and laid eggs containing large amounts of hIFN-β in the egg white. Furthermore, the concentration of hIFN-β in the egg white (denoted KI egg white) of different individuals and individuals of different generations did not vary drastically, demonstrating that, in general, knocking in a transgene at the *OVA* locus could effectively produce chickens that could serve as a bioreactor for mass production of recombinant proteins.

## Results

### Production of the KI chickens

**Figure 1:**
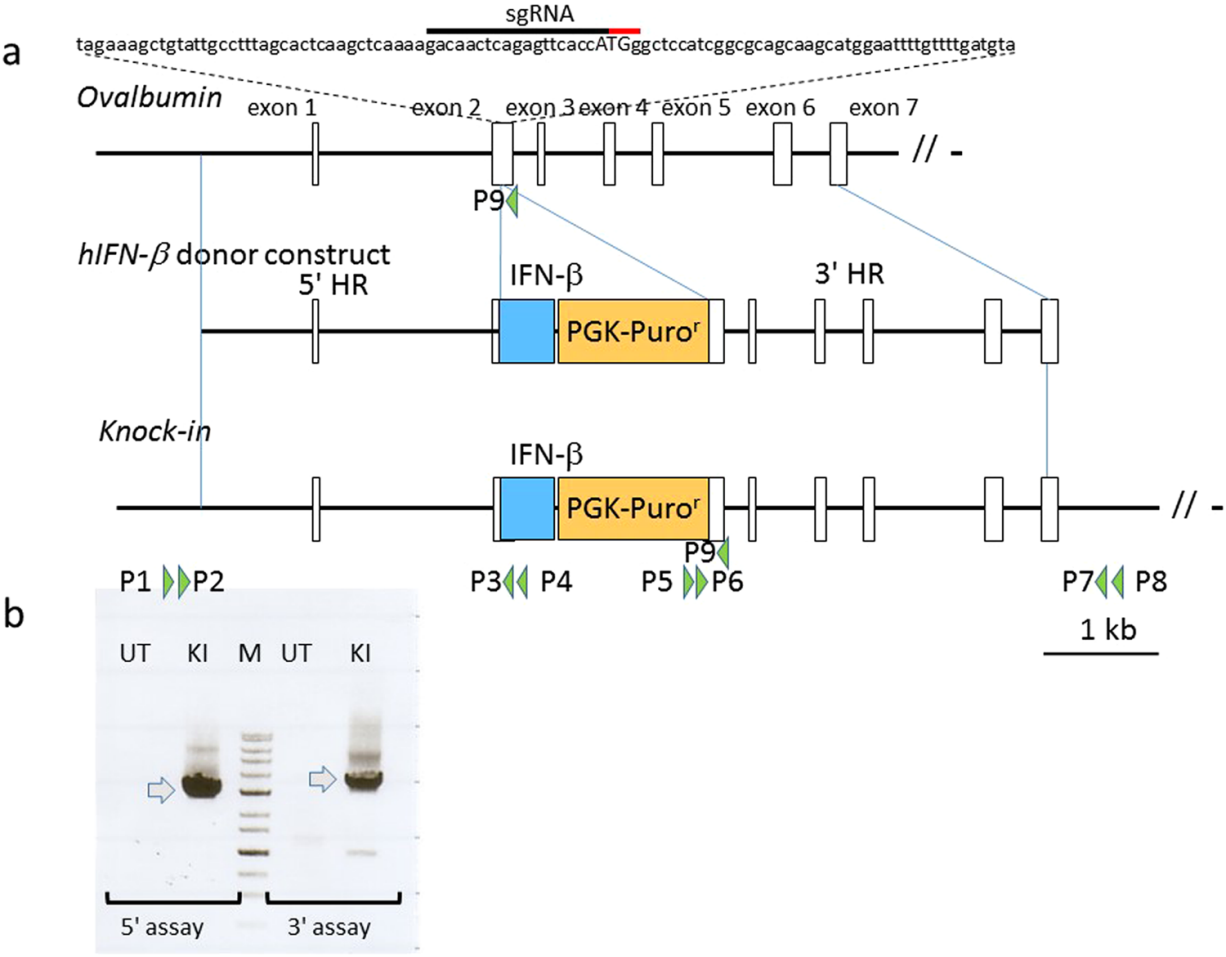
CRISPR/Cas9-mediated human *IFN-*β knock-in at the *OVA* locus in chicken PGCs. (**a**) Schematic of the knock-in strategy. The top diagram shows the WT chicken *OVA* locus. The target single-guide RNA (sgRNA) sequence that is part of exon 2 is denoted by the black bar above the nucleotide sequence. The protospacer adjacent-motif sequence is indicated by the red bar. The *OVA* initiation codon is shown in uppercase letters. The middle diagram shows the donor construct containing the 5′ and 3′ homology regions (HR), the *hIFN-*β -bovine growth hormone polyadenylation signal construct, and the PGK promoter that drives the puromycin resistance gene (PGK-Puro^r^). The bottom diagram shows the KI allele along with PCR primers P1 to P9 that were used for 5′, 3′, and endogenous *OVA* assays in this study. (**b**) PCR amplification of the donor cassette knock-in at the *OVA* locus in the PGC genome. KI PGCs and their parental untransfected cells (UT) were subjected to nested PCR using primers P1–4 (5′ assay) and P5–8 (3′ assay). The middle lane, labeled M, contains DNA molecular mass markers (1-kbp DNA ladder, Nacalai). PCR amplicons of the expected sizes (2.8 kb for the 5′ assay, and 3.2 kb for the 3′ assay) are indicated by the arrows.

To abundantly, stably, and specifically produce recombinant hIFN-β in chicken egg white, we inserted *hIFN-*β at the translation initiation site of the chicken *OVA* locus using a CRISPR/Cas9 system. We had previously constructed and evaluated four sgRNA/Cas9 (sg, single guide RNA) plasmids that could target *OVA* after cell transfection^38^. Based on those results, for the experiment reported herein, we employed pX330-Neo-OVATg1 that targets *OVA* near its transcription initiation codon (Fig. 1a). For insertion of *hIFN-*β, we constructed a donor vector that contained a 5′ *OVA* homology arm of 2.8 kb, the *hIFN-*β coding region fused to the *OVA* initiation codon, the bovine growth hormone polyadenylation signal, the puromycin resistance gene sequence, and a 3′ *OVA* homology arm of 3.2 kb. The donor and pX330-Neo-OVATg1 vectors were co-transfected into PGCs isolated from Barred Plymouth Rock chicken (BPR) male embryos. PGCs containing the donor plasmid were selected with puromycin (0.5 μg/ml). Then, these cells were expanded, and genomic DNA from a portion of the cells was subjected to PCR to confirm that the cells had been knocked in as designed. Using the 5′- and 3′- primer pairs, namely P1–4 and P5–8, located outside the homology regions but inside the transgene (Fig. 1a), the 2.8-kb and 3.2-kb fragments were amplified for the 5′ and 3′ assays, respectively (Fig. 1b; see Supplementary Table S1). The results of these assays indicated that *hIFN-*β had been knocked in at the *OVA* locus in at least some of the cells.

**Figure 2:**
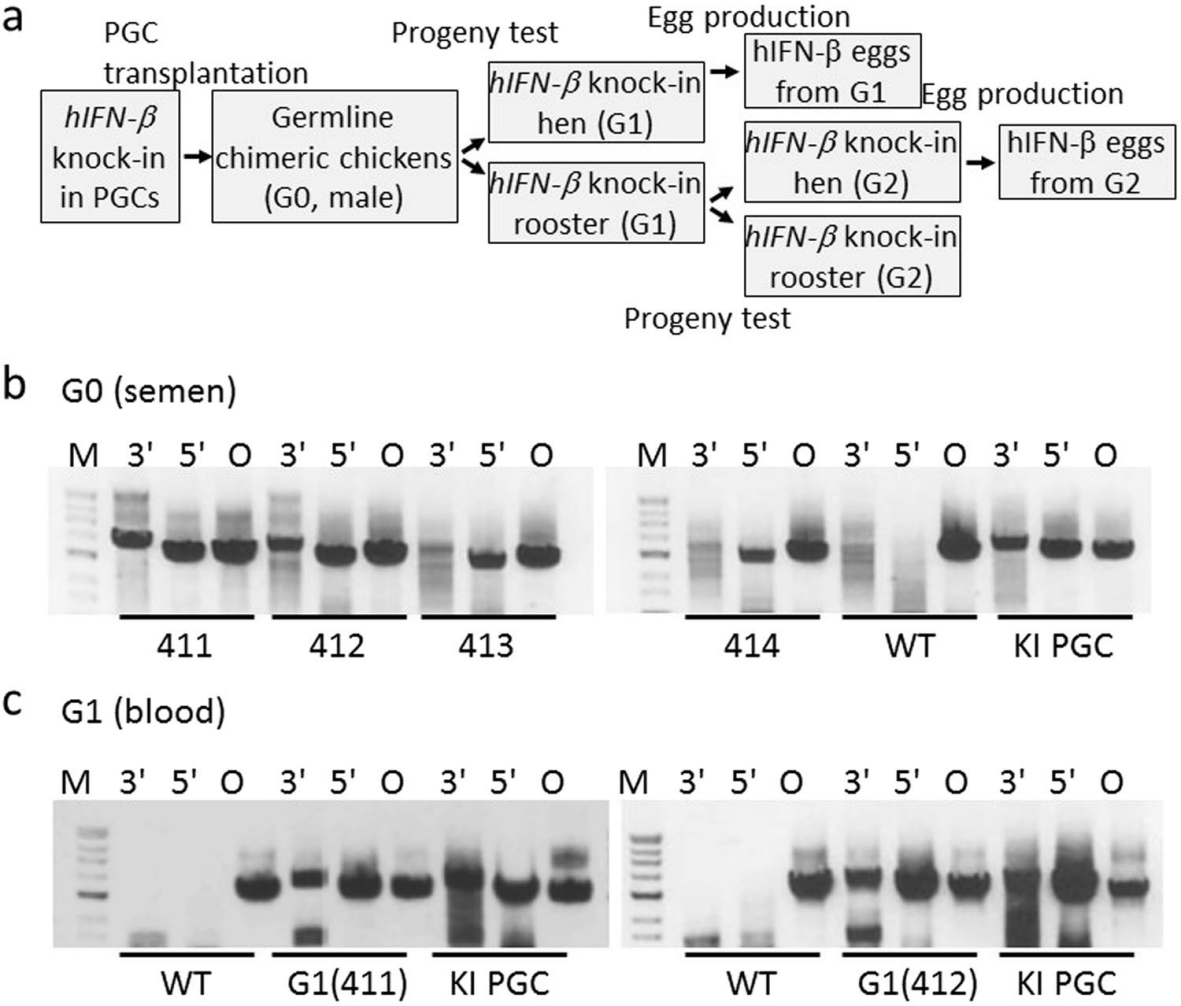
CRISPR/Cas9-mediated knock-in of *hIFN-*β at the chicken *OVA* locus. (**a**) Schematic of the experimental procedure that generated *hIFN-*β KI chickens. (**b**) KI *hIFN-*β in semen of chimeric G0 roosters. Genomic DNA from the sperm of four chimeric roosters (411–414) and a WT rooster was PCR amplified with primer pairs P5/P8, P1/P4, and P1/P9 for the 3′ and 5′ assays and the endogenous *OVA* assay (O), respectively. Genomic DNA from transplanted PGCs containing *hIFN-*β KI cells (KI PGC) was also PCR amplified. The gels show the PCR-amplified products. (**c**) KI *hIFN-*β in the G1 chickens. Genomic DNA from the blood of the G1 progenies of #411 (left panel) and #412 (right panel) was PCR amplified for the 3′, 5′, and endogenous *OVA* assays using primer pairs P5/P8, P1/P4, and P1/P9, respectively. The genomic DNA from the blood of WT chickens and from the transplanted PGCs (KI PGC) was also PCR amplified. The gels show the PCR-amplified products. The lanes at the left of each gel panel are the DNA molecular mass markers as described in Fig. 1.

We next determined whether the KI PGCs could become functional spermatozoa. For this purpose, we generated germline G0 chimeras by transplanting KI cells into the blood of recipient chick embryos at day 2.5 of development (Fig. 2a). To increase the contribution of the donor PGCs, endogenous PGCs in the recipient embryos were ablated by exposing them to 5–6 Gy of γ-radiation before transplantation^38^. After being injected with the KI cells, the recipient embryos were incubated until hatching. Four presumptive male germline G0 chimeras (#411–414) were raised to sexual maturity, and genomic DNA from their semen was subjected to PCR to determine whether their semen contained donor PGC-derived *hIFN-*β KI cells. The semen of all four roosters contained *hIFN-*β KI cells (Fig. 2b). The two roosters (#411 and #412) that showed relatively strong KI PCR products by both the 5′ and 3′ assays were crossed with wild-type (WT) White Leghorn (WL) hens. The genotypes of their G1 progeny were also assessed by PCR (Fig. 2b), and the results showed that 22.5% and 14.5% of the progeny from roosters #411 and #412, respectively, had the *hIFN-*β knock-in at the *OVA* locus (Table 1). We obtained 15 male and 16 female KI G1 chicks, and all were normal in appearance and had no discernable growth defects.

**Table 1:** Efficiency of OVA targeting in G1 offspring.

parent (G0)	No. of hatched chicks	No. of knock-in chicks	No. of knock-in male chicks	No. of knock-in female chicks	% of knock-in offspring
#411	102	23	12	11	22.5
#412	55	8	3	5	14.5

### Production of recombinant hIFN-β in the oviduct of KI hens and its deposition into egg white

**Figure 3:**
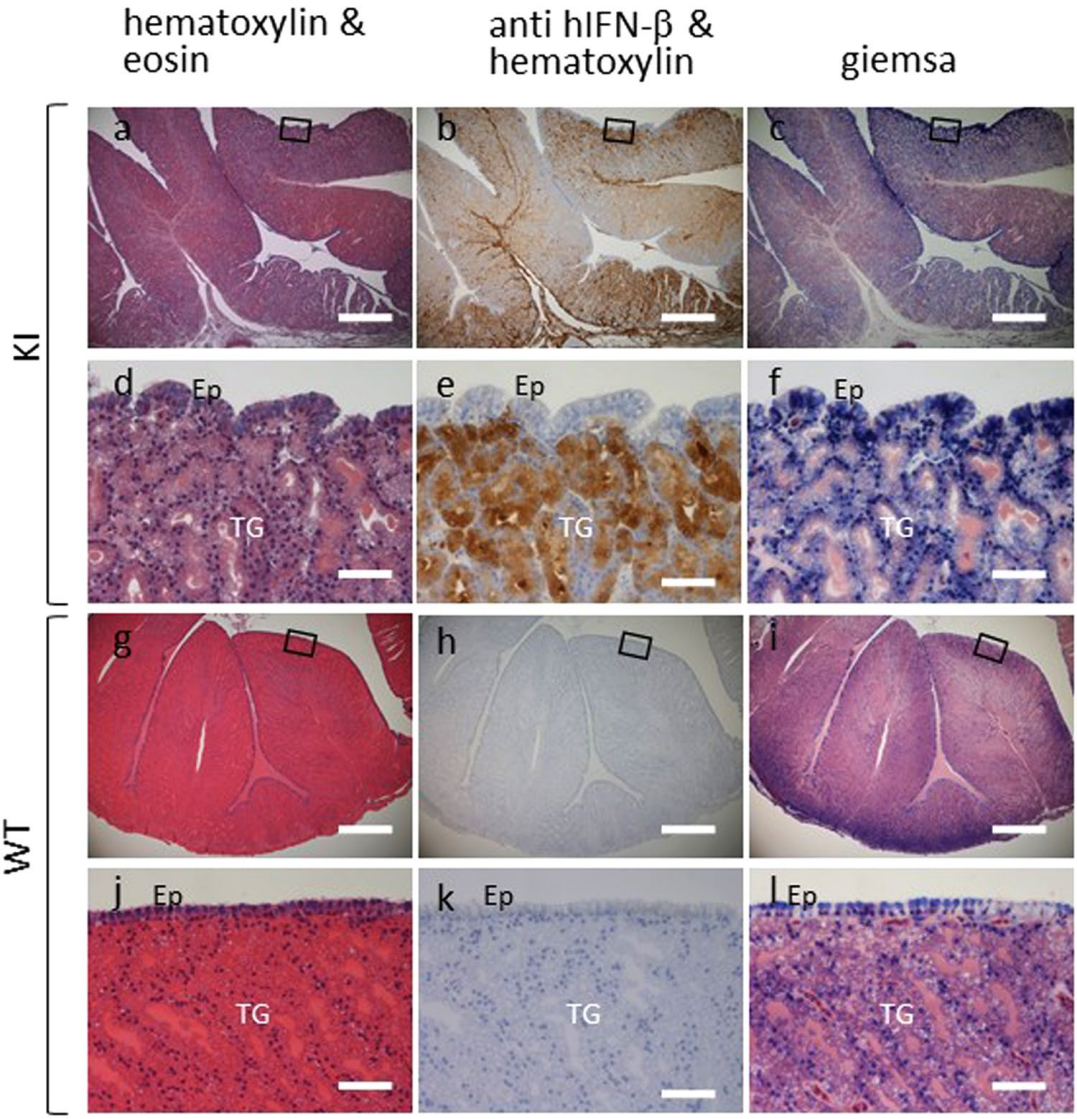
Immunohistochemistry of hIFN-β in the oviduct magnum sections. Oviduct sections from *hIFN-*β KI (**a**–**f**) and WT (**g**–**l**) hens were stained with hematoxylin and eosin (**a**,**d**,**g** and **j**), immunohistochemically stained for hIFN-β and counterstained with hematoxylin (**b**,**e**,**h** and **k**), or stained with Giemsa (**c**,**f**,**i** and **l**). Panels d–f and j–l are magnified views of the enclosed rectangular sections in panels a–c and g–i, respectively. The presence of hIFN-β is apparent in the oviduct magnum section from the *hIFN-*β KI hen (**b**,**e**) with its expression restricted to the tubular glands (TG). Ep, epithelial cells. All sections were counterstained with hematoxylin. Scale bars, 500 μm (**a**–**c**,**g**–**i**); 50 μm (**d**–**f**,**j**–**l)**.

Expression of endogenous *OVA* is restricted to the tubular gland cells in the oviduct magnum^39^. To determine the site of ectopic gene expression in our KI hens, we performed immunohistochemistry for hIFN-β on sections of the oviduct magnum (Fig. 3). Consistent with endogenous *OVA* expression, hIFN-β was found in the tubular gland cells of the KI hens but was absent in adjacent epithelial cells (Fig. 3c), indicating that hIFN-β expression was controlled by *OVA* regulatory mechanisms. Expression of hIFN-β was not detected in the oviduct magnum of WT WL hens (Fig. 3e and f).

**Figure 4:**
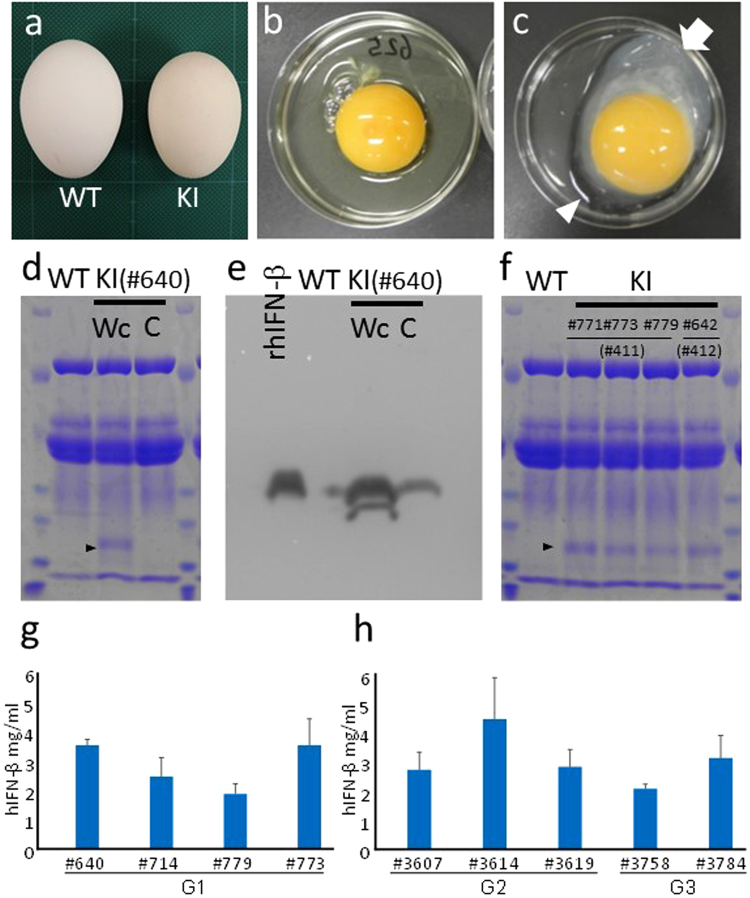
Production of recombinant hIFN-β in KI egg white. (**a–c**) Eggs from a *hIFN-*β KI and a WT hen. (**a**) Eggs from WT and KI hens. (**b**,**c**) The white and yolk from an egg of a WT (**b**) or KI (**c)** hen. Most of the KI egg white appeared to be white and cloudy (arrow in **c**). The arrowhead in (**c**) indicates a clear section of the egg white. (**d**) Production of hIFN-β in the white of eggs from a KI (#640) and WT hen as assessed by SDS-PAGE. The arrowhead indicates the position of hIFN-β. Wc, white, cloudy part of the KI egg white; C, clear part of the KI egg white. (**e**) Immunoblotting of KI egg-white proteins with anti-hIFN-β. rhIFN-β, recombinant hIFN-β (40 ng); WT, Wc, and C have the same meanings as in Fig. 4d. For each lane, 3 μg of the egg white was electrophoresed. (**f**) SDS-PAGE showing similar production levels of hIFN-β in the white of eggs laid by the four KI hens (#771, #773, #779, #642). The two parental G0 KI roosters (#411, #412) of the hens are identified in the panel. (**g, h**) Concentration of recombinant hIFN-β in KI egg white from G1, G2, and G3 KI hens. Solutions of the cloudy part of the KI egg white from four G1 hens (**g**), three G2 hens, and two G3 hens (**h**) were subjected to ELISA for the presence of recombinant hIFN-β. Bars show the mean ± standard deviation of the concentration of recombinant hIFN-β in each KI egg white. Four (G1) and three (G2 and G3) eggs from individual KI hens were subjected to ELISA.

Next, we studied the effect of the hIFN-β knock-in on egg production. The average production rate for the G1 KI hens was about two-thirds that of the WT controls (BPR × WL) and was statistically significant (p < 0.05, Welch’s *t*-test; Table 2). In addition, all G1 hens produced eggs, although their eggs appeared to be smaller than those of WT hens (Fig. 4a). Consistent with this observation, the average weight of the eggs from 10-month-old KI hens was 42.1 ± 3.1 g (n = 26), which was significantly less than that of WT (BPR × WL) (50.1 ± 3.4 g (n = 16); p < 0.05, Student’s *t*-test). Unexpectedly, the portion of the KI egg white closest to the yolk was white and cloudy (Fig. 4b and c), whereas the periphery portion of the egg white was clear and apparently of low viscosity. The volume of the clear region was relatively small compared with that of the cloudy region (4.3 ± 1.8 ml/egg vs. 14.4 ± 3.6 ml/egg, respectively, n = 41).

**Table 2:** Egg production by hIFN-β KI G1, G2, and G3 hens and by WT (G1) hens.

Chickens	Bird no.	Parental rooster	Age at first egg (day)	First 150 days egg production (no.)	Egg production rate (day^–1^)	Duration of life span (days)
Knock-in G1	#640	#411	146	58	38.7	615
(BPR × WL)	#714	#411	131	121	80.7	993
	#766	#411	136	21	14.0	979
	#773	#411	129	96	64.0	775
	#779	#411	138	103	68.7	950
	#813	#412	159	53	35.3	535
Average value			139.8 ± 10.2	75.0 ± 34.8^*^	50.0 ± 23.2^*^	807.8 ± 181.0
Control G1	#C1		105	115	76.7	985
(BPR × WL)	#C2		129	110	73.3	818
	#C3		129	117	78.0	945
	#C4		129	114	76.0	818
Average value			123 ± 10.4	114.0 ± 2.5	76.0 ± 1.7	891.5 ± 74.8
Knock-in G2	#3058	#613 (G0:#411)	146	38	25.3	
	#3059	#613 (G0:#411)	146	79	52.7	
	#3063	#731 (G0:#412)	150	67	44.7	
	#3602	#534 (G0:#412)	138	23	15.3	
	#3607	#731 (G0:#412)	132	133	88.7	
	#3610	#731 (G0:#412)	127	11	7.3	
	#3614	#767 (G0:#411)	127	107	71.3	
	#3619	#782 (G0:#411)	159	63	42.0	
Average value			140.6 ± 10.8	65.1 ± 38.7	43.4 ± 25.8	
Knock-in G3	#3758	#3604(G0:#412)	142	147	98.0	
	#3781	#3604(G0:#412)	132	44	29.3	
	#3784	#3604(G0:#412)	132	52	34.7	
Average value			135.3 ± 4.7	81.0 ± 46.8	54.0 ± 31.2	

^*^Significantly different compared with control, as calculated by a Weltch’s t-test (P < 0.05).

Next, we determined whether the KI egg white contained recombinant hIFN-β. The cloudy and clear parts of the KI egg white (from the #640 KI hen) as well as the thick albumen from the egg of a WT (BPR × WL) hen were subjected to SDS-PAGE followed by Coomassie Brilliant Blue staining (Fig. 4d). A Coomassie Blue–stained band for protein from the cloudy part of KI egg white was observed near that of the expected size (~23 kDa) for hIFN-β^40^. The same band was nearly absent in the clear part of the KI egg white and was not seen at all in WT egg white. We also subjected the egg-white samples to immunoblotting with an antibody against hIFN-β. Consistent with the Coomassie Blue staining results, substantial and relatively weak putative IFN-β signals were detected in the samples of the cloudy and clear portions of the KI egg white, respectively (Fig. 4e). In contrast, no obvious band of the same mass as hIFN-β was observed in the egg white from the WT hen. Based on results from both SDS-PAGE and immunoblotting, it was evident that hIFN-β was deposited in KI egg white, although not evenly distributed as it had accumulated in the cloudy part rather than in the clear part. The egg-white proteins from eggs of four additional KI G1 hens were also subjected to SDS-PAGE (Fig. 4f), and the cloudy portion of each KI hen egg white contained substantial and similar amounts of hIFN-β, which indicated that insertion of *hIFN-*β into the *OVA* locus was precisely controlled by the *OVA* transcriptional regulatory machinery and that hIFN-β was almost equally expressed in the oviduct of the KI hens and then secreted into their egg white.

We sequenced the first five *N*-terminal residues of hIFN-β from a KI egg white. Although the coding region of *hIFN-*β, including its signal sequence, was introduced into the *OVA* locus, the *N*-terminal sequence of hIFN-β from the KI egg white was that of mature hIFN-β expressed in mammalian cells (NH_2_-MSYNL), suggesting that the signal sequence had been properly cleaved^41^. The concentrations of hIFN-β in the KI egg white from four eggs each from four G1 hens was measured by an enzyme-linked immunosorbent assay (ELISA). The hIFN-β concentration in the cloudy part and clear part of the egg white ranged from 1.86 ± 0.34 to 3.52 ± 0.89 mg/ml and from 0.14 ± 0.05 to 0.68 ± 0.19 mg/ml, respectively (Fig. 4g). We also measured the hIFN-β concentration in chicken serum. As shown in Supplementary Table S2, hIFN-β was detected in serum from KI hens (from 0.23 ± 0.04 to 1.75 ± 0.12 ng/ml) but not in serum from a KI rooster, suggesting that portion of the hIFN-β expressed in the oviduct was transferred into the bloodstream.

Next we assessed the fertility of the G1 KI hens. These hens were crossed with a WT WL rooster, and the resulting eggs were incubated; however, all fertilized eggs did not develop past day 8 so that no hatched chicks were obtained (n = 34). To determine why the eggs from hIFN-β KI hens were sterile, we transferred a portion of the KI or WT egg white into WT fertilized eggs and incubated them. Indeed, the presence of hIFN-β KI egg white significantly disrupted the embryonic development of recipient eggs (Supplementary Fig. 1; p < 0.05, chi-square test). This result indicated that the hIFN-β KI egg white was detrimental to the development of the chicken embryo, although it was unclear whether this was the only underlying cause of the egg sterility.

To obtain G2 offspring KI hens, we crossed a G1 KI rooster with WT WL hens. Unlike crossing the G1 KI hens with WT roosters, the crosses involving the KI roosters and WT hens produced male and female G2 KI progeny. The G2 KI hens were raised to sexual maturity, and their ability to lay eggs was assessed. Although, the G1 and G2 hens did not share exactly the same genetic background, the egg production rate and age at first egg laid did not differ significantly between the G1 and G2 groups (Table 2). Furthermore, the hIFN-β concentration in the cloudy part of the egg white was an inheritable feature (2.70 ± 0.59 to 4.42 ± 1.40 mg/ml, Fig. 4h). Both the efficiency of egg production and hIFN-β concentration in cloudy part of the egg white were similar in the G1 and G2 hens, which suggested that the average hIFN-β productivity of the KI hens was not drastically affected by the generation, at least if they had similar genetic backgrounds. Consistent with this idea, the mean values for egg production and hIFN-β concentration were also similar among G1, G2, and G3 (2.06 ± 0.15 and 3.10 ± 0.76 mg/ml, Fig. 4h) hens, which were generated by crossing with a G2 male and WT WL hens (Table 2 and Fig. 4h).

### Characterization of hIFN-β produced by the KI hens

**Figure 5:**
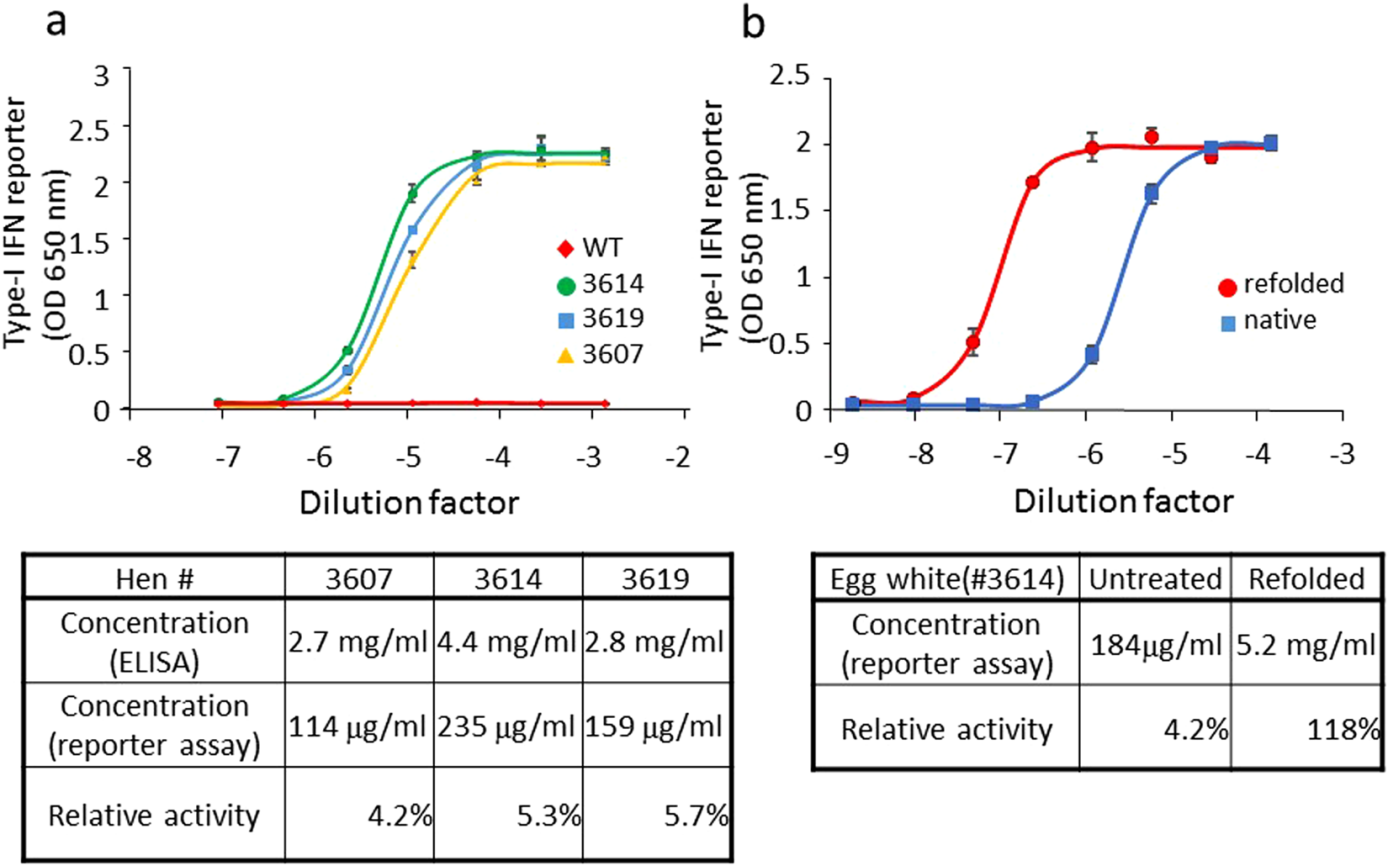
Detection of bioactive hIFN-β in KI egg white. (**a**) HEK-Blue IFN-α/β reporter cell samples were individually stimulated with five-fold serial dilutions of egg white from three *hIFN-*β KI hens (#3607, #3614, #3619) and a WT hen. The cell culture supernatants were assayed for induction of secreted alkaline phosphatase reporter activity using a colorimetric assay. Plots show the mean ± standard deviation values for three independent assays. A four-parameter logistic curve was fit to each dataset, and the EC_50_ values (median effective concentrations) were calculated for each curve (upper panel). By using the EC_50_ of a commercially available, purified recombinant hIFN-β as the standard, the absolute IFN-β bioactivities in the KI egg white were calculated [shown as “Concentration (reporter assay)” in the table at the bottom panel]. For comparison, the concentration of hIFN-β in each egg white was determined by ELISA (Fig. 4h), and the relative hIFN-β bioactivities were calculated as the percentage of active hIFN-β in the total amount of IFN-β. (**b**) Egg white from the *hIFN-*β KI hen #3614 was denatured in 6 M guanidine hydrochloride and then renatured in the presence of the artificial chaperone, highly polymerized cycloamylose (see Methods). hIFN-β bioactivity in the untreated and renatured egg white was analyzed by the HEK-Blue IFN-α/β reporter assay. Plots show the mean ± standard deviation values for three independent assays. The data are reported as in Fig. 5a.

Given the abundant deposition of hIFN-β in the KI egg white, we next examined whether the deposited hIFN-β had biological activity. The cloudy part of the egg white from three eggs produced by each of three different KI hens and the thick albumen from an egg of a WT hen were sonicated, serially diluted with phosphate-buffered saline (PBS), and assayed for hIFN-β bioactivity by a reporter assay that used HEK-Blue IFN-α/β reporter cells to induce production of secreted alkaline phosphatase following stimulation with human type-I interferon^42^. The secreted alkaline phosphatase was quantified for each sample, and their half-log dose response curves were plotted (Fig. 5a, top panel). All KI egg white exhibited IFN bioactivity, but the egg white from the WT hen did not. However, the relative bioactivity of IFN-β in the KI egg whites was 4.2–5.7% that of commercially available, purified recombinant hIFN-β produced in mammalian cells (Fig. 5a, bottom table). A possible reason for this reduced bioactivity was that the majority of the protein was aggregated and/or misfolded. To address this possibility, we treated KI egg white of the KI hen #3614 with 6 M guanidine hydrochloride to ensure that the hIFN-β was unfolded; we then refolded the protein by dilution into a solution containing an artificial chaperone, namely highly polymerized cycloamylose^43^. The bioactivity of hIFN-β in both untreated and treated KI egg white was then examined. As shown in Fig. 5b, the hIFN-β activity after unfolding and refolding was >28-fold greater than that in the untreated egg white and was regarded as fully recovered to the level of that measured for commercially produced hIFN-β. This result indicated that the majority of hIFN-β in the KI egg white was misfolded and/or aggregated and hence was inactive, but activity could be restored upon refolding.

## Discussion

This study reports the first successful knock-in of a gene in chickens for the production of an encoded recombinant protein in their eggs. All analyzed G1 and G2 KI hens produced large amounts of recombinant hIFN-β in the white of their eggs (1.86–4.42 mg/ml; ~30–60 mg (~1.3–2.7 μmol) per egg), demonstrating that gene targeting in chickens represents a potentially powerful means of producing recombinant proteins. Average egg production was ~70 per hen among the KI hens within the first 150 days (Table 2), implying that <500 KI hens could provide a kilogram of recombinant hIFN-β in this time frame.

To date, lentiviral vectors have been the preferred transgene vehicle for the production of transgenic chickens because they efficiently modify germlines^22^. Lillico and colleagues generated a transgenic hen line using a lentiviral vector containing the 5′ regulatory sequence of *OVA* that included putative regulatory elements and *hIFN-*β^16^. As with our KI hens, the lentivirus-mediated G1 and G2 transgenic hens specifically expressed *hIFN-*β in their oviduct and secreted bioactive recombinant hIFN-β into the egg white. Unlike our system, however, the average concentration of hIFN-β in the white of eggs from the G1 and G2 lentiviral transgenic hens (3.5–426 μg/ml; the range is the largest and smallest mean value calculated for six individual hens) varied greatly and was relatively less than that from our hens (1.86–4.42 mg/ml). The different results for the two hIFN-β systems may be a consequence of position effects, although other factors, e.g., genetic background and the physical conditions of the hens, may also have contributed. In addition, the difference between endogenous *OVA* regulatory elements in the KI hens and the short, truncated *OVA* regulatory elements introduced into the lentiviral transgenic hens may have differentially affected transgene expression. Although the precise regulatory mechanism(s) controlling expression remains to be clarified, our knock-in system resulted in stable expression of h*IFN-*β and production of large amounts of the recombinant protein, which suggests that ours would be the preferred system for commercial production of recombinant proteins.

The eggs laid by our KI hens included cloudy egg white (Fig. 4c). Given that hIFN-β accumulated in the cloudy part of the egg white, it is probable that hIFN-β had aggregated (possibly in association with other proteins). Protein aggregation is caused by various factors including misfolding, an excessive concentration, and physical parameters such as temperature, pH, and ionic strength^44^. The observed reduced bioactivity of hIFN-β in the KI egg white suggests that most of the hIFN-β molecules had misfolded and/or aggregated (Fig. 5a). Consistent with this idea, the specific bioactivity of hIFN-β in the egg white was drastically increased (and appeared to be completely recovered) after subjecting the KI egg white to the unfolding-refolding procedure (Fig. 5b). Because refolding of hIFN-β in the egg white was easily achieved, aggregation could be an advantageous property of recombinant proteins produced in our KI chicken system, i.e., it could be used to at least partially isolate hIFN-β or another aggregated recombinant protein by centrifugal enrichment of the cloudy aggregate for subsequent refolding and activation.

The observed aggregation of hIFN-β may have been a consequence of its high concentration in the egg white. Various exogenous proteins have been expressed in transgenic chickens; however, aggregation of these proteins in egg white has neither been reported nor, apparently, studied in depth. Even for the scFv-Fc transgenic hen system, for which scFv-Fc was produced in greater quantities than hIFN-β in our system, the physical state of the egg white containing scFv-Fc was not reported^21^. Therefore, it remains to be determined if aggregation of a protein in the white of eggs from transgenic hens can be fully attributed to the concentration of the protein or, at least in part, to the specific characteristics of the protein. To differentiate among the underlying physical properties that can induce aggregation, in addition to hIFN-β, the folding of other proteins in egg white should be analyzed by generating oviduct-specific gene-targeted hens. These studies would increase our understanding of the mechanism underlying the control of secretion of an expressed foreign protein into KI egg white. Moreover, methods must be developed to avoid protein aggregation and misfolding for the production of large-sized proteins and protein complexes, each of which is not suited to protein refolding.

Protein *N*-glycosylation plays important roles in protein folding, stability, and function^45^. Human IFN-β is an *N*-glycosylated protein, and a non-glycosylated form produced by *Escherichia coli* was found to have substantially less antiviral activity and indeed formed inactive dimers and oligomers^46^. It has been reported that the recombinant proteins produced in egg white are *N*-glycosylated, but they lack galactose and sialic acid in the *N*-glycans^13,14,47^. This glycosylation pattern is mainly attributable to low expression of galactosyltransferase in the oviduct magnum^48^, and thus the *N*-glycans of hIFN-β deposited in egg white may have lacked the terminal galactose and sialic acid. Although a detailed analysis of glycosylation of hIFN-β in KI egg white would be required to address this issue, the lack of addition of these two terminal sugars may underlie our observed aggregation of hIFN-β in egg white as well as other possible alterations such as a reduction in protein half-life^13^. In this respect, transgenic chickens that ectopically express galactosyltransferase may constitute a platform to generate a bioreactor system based on KI hens^49,50^. Hence, proper galactosylation followed by sialyation in the oviduct magnum might resolve the issue of protein aggregation in egg white.

Our KI chickens were normal in appearance and had no obvious growth or health problems. In addition, total life span did not differ significantly between KI and control hens (Table 2, G1). Conversely, both the number and size of the KI hen eggs were reduced compared with WT (Table 2 and Fig. 4a). Because IFN-β induces various biological reactions such as antiviral and antiproliferative responses and may cause toxicity and infertility^51,52^, and thus it is plausible that a large amount of hIFN-β in the oviduct gland cells and/or the oviduct lumen negatively affected egg production. Although we did not observe any apparent histological abnormalities in the oviduct of the KI hens (Fig. 3), it is possible that the oviduct cells experienced damage and/or malfunction. The presence of hIFN-β in blood of KI hens (Supplementary Table S2) may also have contributed to the reduction in egg production. Moreover, heterozygous mutation of *OVA* might have negatively impacted egg production owing to the reduced production of OVA protein. In this respect, employment of a targeting vector, i.e., one designed to not affect endogenous OVA expression using a self-cleaving peptide or bicistronic expression system, might result in normal egg production as well as an abundance of recombinant protein.

As shown in this study, even though the KI hens were sterile possibly owing to foreign protein expression, the fertility of the KI roosters was hardly affected. Roosters are very fertile; therefore, in principle, the sperm from a KI rooster could easily be used to generate >1000 KI male and female offspring by artificial insemination. Each male offspring could then act as a new founder, and the female offspring would stably produce large quantities of a recombinant protein in egg white, allowing for large-scale production of the protein within a few generations of the first KI rooster. This scalability and time-efficient expansion of a KI bioreactor are advantages that chickens have compared with other types of livestock (i.e., goats and cows) and plant bioreactor systems. In addition, because transgenic hen systems can be developed easily, expression of a foreign protein in the eggs of transgenic hens is expected to be stable over time, gene targeting at the *OVA* locus has the potential to be the preferred technique to establish transgenic chicken bioreactors and the key driver for mass production of recombinant proteins using chickens.

## Methods

### Animal experiments

All animal experiments were conducted according to protocols approved by the institutional animal care and use committees of the National Institute of Advanced Industrial Science and Technology and the National Agriculture and Food Research Organization Institute of Livestock and Grassland Science. The WL and BPR chickens were maintained at the animal farm facility of the National Agriculture and Food Research Organization Institute of Livestock and Grassland Science.

### Plasmid construction

The plasmid expressing hCas9 and sgRNA targeted to *OVA* (px330-Neo-OVATg1) were generated as described elsewhere^38^. The donor vector for hIFN-β was generated by ligating a PCR-amplified, 2.8-kb DNA *OVA* fragment (upstream of the ATG initiation codon) as the 5′ homology arm, the cDNA containing the encoded hIFN-β sequence, the bovine growth hormone polyadenylation and puromycin resistance gene sequences, and a PCR-amplified, 3.2-kb DNA an *OVA* 3.2-kb sequence as the 3′ homology arm at the *Sal*I and *BamH*I sites in pBluescript II SK(+). Primers for PCR amplification are shown in Supplementary Table S1, and PCR was carried out with PrimeSTAR HS DNA polymerase (TaKaRa, Otsu, Japan).

### Targeted gene knock-in of cultured PGCs

PGCs derived from the blood of BPR embryos at Hamburger-Hamilton stages 14 to 16^53^ were cultured and transfected with px330-Neo-OVATg1 and the *hIFN-*β donor vector using Lipofectamine 2000 (Invitrogen, Carlsbad, CA), as described previously^38^. Briefly, 0.8 μg each of px330-Neo-OVATg1 and the *hIFN-*β donor vector diluted with 50 μl OPTI-MEM (Thermo Fisher Scientific, Waltham, MA) was mixed with 50 μl OPTI-MEM containing 3 μl Lipofectamine 2000 reagent and then incubated with 0.5–1 × 10^5^ PGCs for 5 min. Subsequently, the cells were suspended in 400 μl of an antibiotic-free KO-DMEM-type culture medium (Thermo Fisher Scientific) and incubated for 2 h at 37 °C^38^. PGCs were the cultured with Buffalo rat liver feeder cells for 4 days and then selected with puromycin (0.5 μg/ml; InvivoGen, San Diego, CA) for 3 days. The selected PGCs were expanded for 2 to 3 weeks and then selected again with puromycin under the same conditions. Finally, integration of *hIFN-*β at the *OVA* locus was confirmed by PCR using genomic DNA isolated from a proportion of the cells (see below).

### Detection of *hIFN-*β in the *OVA* locus

Genomic DNA was extracted from PGCs, from the semen of G0 roosters, and blood from G1 and G2 roosters and hens using reagents of the DNeasy Blood and Tissue kit (Qiagen, Valencia, CA). Gene-targeting events were detected by single or nested PCR using MightyAmp DNA polymerase Ver.2 (TaKaRa) with the primers shown in Supplementary Table S1.

### Generation of germline G0 chimera

Fertilized eggs for recipient embryos were irradiated with γ-rays at 5 to 6 Gy using a Gammacell 40 irradiator (Atomic Energy of Canada Ltd., Chalk River, ON, Canada). PGC samples (1000–2000 cells) that included the transgenic cells, were injected into the bloodstream of the recipient embryos (Hamburger and Hamilton stages 13–15). The male embryos, identified by PCR^54,55^, were incubated until they hatched as described elsewhere^38^ and then were raised to sexual maturity, after which genomic DNA from sperm was analyzed with PCR.

### KI egg analyses

A disposable medical dropper was used to collect samples of the clear and cloudy parts of the KI egg white based on their apparent viscosities and appearances. The volumes of the samples were then measured using the scales on the sides of the tubes. The viability of the embryos derived from the KI hens was assessed by candling the eggs on days 5 and 10. Dead embryos at the various developmental stages were confirmed by breaking eggs and examining the embryos.

### Analysis of hIFN-β in the KI egg white

Sonicated samples of KI and WT egg white were individually suspended in 62.5 mM Tris-HCl, pH 6.8, 1% (w/v) SDS at a ratio of 1:20 for SDS-PAGE. Samples were each mixed with an equal volume of 2× Laemmli SDS-PGE sample buffer and subjected to gradient SDS-PAGE (5–20% w/v acrylamide; Oriental Instruments Ltd. Tokyo, Japan). Each gel was then stained overnight with Coomassie Brilliant Blue R-250 (Nacalai, Kyoto, Japan). The stained band from each lane corresponding to the molar mass of hIFN-β was excised and subjected to *N*-terminal sequencing (Hokkaido System Science Co., Ltd., Sapporo, Japan).

For immunoblotting, egg-white proteins (3 μg per lane) and recombinant hIFN-β (40 ng per lane, Wako, Osaka, Japan) were separated by SDS-PAGE and transferred onto a polyvinylidene difluoride membrane (Immobilon P; Millipore, Bedford, MA). The membrane was subjected to immunoblotting with anti-hIFN-β (ab85803; Abcam, Cambridge, UK) at 1 μg/ml and visualized with horseradish peroxidase–conjugated anti-rabbit IgG (Jackson ImmunoResearch, West Grove, PA) using an enhanced chemiluminescence system (ImmunoStar reagent; Wako).

The concentration of hIFN-β in egg white was measured by ELISA (VeriKine Human Interferon Beta ELISA kit; PBL Assay Science, Piscataway, NJ). Chinese hamster ovary cell–derived recombinant hIFN-β (Wako) suspended in 62.5 mM Tris-HCl, pH 6.8, 1% (w/v) SDS served as the standard.

### hIFN-β reporter assay

Bioactive hIFN-β produced in KI egg white was detected using HEK-Blue IFN-α/β reporter cells (InvivoGen). In brief, sonicated egg white was serially diluted with PBS, and then 20 μl of each dilution was added onto HEK-Blue IFN-α/β reporter cells: 5 × 10^4^ cells per well in 180 μl of a DMEM-based culture medium (Thermo Fisher Scientific) in 96-well plates. After culture overnight at 37 °C, 20 μl of the culture supernatant from each sample was added into 180 μl Quanti-Blue reagent (InvivoGen) and then incubated for 1 h at 37 °C. The activity of the secreted alkaline phosphatase was measured as a colorimetric reaction at 650 nm using a microplate reader (Emax plate reader, Molecular Devices, Sunnyvale, CA). Results were analyzed using the Curve Fitter program of ImageJ (NIH, Bethesda, MD) to calculate half-maximal effective concentration (EC_50_) values for hIFN-β from egg white and for the Chinese hamster ovary cell–derived recombinant hIFN-β (Wako).

### Unfolding and refolding of hIFN-β

Unfolding and refolding of recombinant hIFN-β in KI egg white was performed with the Refolding CA kit (Takara). In brief, the egg white was sonicated and diluted 1:10 with PBS, then unfolded in 6 M guanidine hydrochloride with 40 mM dithiothreitol (final concentrations) for 1 h. The egg-white samples were then suspended in a 70-fold volume of surfactant solution (0.05% v/v Tween 40 and 2 mM dl-cystine in PBS) and incubated for 1 h at room temperature. Proteins were then refolded by adding 0.6% (v/v) of highly polymerized cycloamylose at a final concentration, followed by an 8-h incubation at room temperature. Samples were centrifuged at 20,000 × *g* for 10 min, and the supernatant was used as the refolded protein solution.

### Immunohistochemistry

A KI hen and a WT WL hen were sacrificed when they were 306 and 294 days old, respectively. The middle parts of their oviduct magnum were collected, fixed in 4% (w/v) paraformaldehyde, and then embedded in paraffin wax. Serial sections 5-μm thick were cut. The sections were deparaffinized in xylene, dehydrated through a graded series of ethanol, and treated with 0.3% (v/v) H_2_O_2_ in methanol to inactivate endogenous peroxidase. After washing in PBS, sections were blocked in 5% (v/v) normal goat serum in PBS containing 0.1% (v/v) Tween-20 for 20 min and then incubated overnight at 4 °C with anti-hIFN-β (ab91245, Abcam). The sections were then rinsed three times in the same buffer, incubated with a peroxidase-conjugated anti-rabbit IgG (Histofine Simplestain Max PO; Nichirei, Tokyo, Japan) and then reacted with diaminobenzidine (Nichirei). Sections were counterstained with hematoxylin.

## Electronic supplementary material

Supplementary information
