# Supplementary material for: Efficient production of human interferon beta in the white of eggs from ovalbumin gene–targeted hens
Source: Sci Rep. 2018 Jul 5;8:10203. doi: 10.1038/s41598-018-28438-2 (PMC6033876; doi:10.1038/s41598-018-28438-2)
Supplement: Supplementary file 1 — Supplementary information [file 41598_2018_28438_MOESM1_ESM.pdf]

Supplementary information

Efficient production of human interferon beta in the whites of eggs from ovalbumin gene–targeted hens.

Isao Oishi[1]*, Kyoko Yoshii[1], Daichi Miyahara[2], &Takahiro Tagami[2]

[1]Biomedical Research Institute, National Institute of Advanced Industrial Science and Technology, 1-8-31, Midorioka, Ikeda, Osaka 563-8577, Japan
[2]Animal Breeding and Reproduction Research Division, National Agriculture and Food Research Organization, Institute of Livestock and Grassland Science, 2 Ikenodai, Tsukuba, Ibaraki 305-0901, Japan

*Corresponding author: oishi-i@aist.go.jp

Supplementary Methods

Egg white transfer. Before incubation, fertilized WT WL eggs (recipient eggs) were windowed, and then ~6–8 ml of egg white was removed. Then, 5 ml of thick albumen of WT WL or the cloudy part of IFN-β KI egg white was added via a disposable medical dropper to the recipient eggs. The window of each recipient egg was sealed, and the eggs were incubated for 1 week. The embryonic development of the recipients was assessed visually on days 6 and 7, and the viability of embryo in each group was calculated on day 7.

Table S1 Primers used in this study

Genotyping	Forward[a]	Reverse[a]
5' assay (single and nested 1st)	CTCTTTCTGCAGACTGACATGCATT (P1)	GCTGTAGTGGAGAAGCACAACAGGAGA (P4)
5' assay (nested 2nd)	ACCTGTGGTGTAGACATCCAGCA (P2)	ATTTGGAGGAGACACTTGTTGGTCAT (P3)
3' assay (single and nested 1st)	CAACCTCCCCTTCTACGAGCGGCT (P5)	AGGACCCAGTGGGACAAATCTA (P8)
3' assay (nested 2nd)	ACCGAAAGGAGCGCACGACCCCAT (P6)	CAACTTCTAGGGCCATACCTGCT (P7)
OVA assay	CTCTTTCTGCAGACTGACATGCATT (P1)	AAAATTCCATGCTTGCTGCACCGAT (P9)
Constructtion	**Forward[a]**	**Reverse[a]**
OVA5' 2.8 kb	GTCGACCTTAAGTCCTCAGACTTGGC	AGACACTTGTTGGTCATGGTGAACTCTGAGTTGTC
OVA3' 3.2 kb	TCCATCGGTGCAGCAAGCATGGAATT	GGATCCACATCATCTGCACAGGTTTGCT
human interferon beta cDNA	GACAACTCAGAGTTCACCATGACCAACAAGTGTCT	GTCGACAGAGGCACAGGCTAGGAGATCTTCA
bGH-polyA	AAAGTCGACTCGCTGATCAGCCTCGACT	AAACTCGAGTGCCTGCTATTGTCTTCCCAAT
PGK-puromycin	GTCGACCCTCGACCTCGAAATTCTACC	TGCAGCTTATAATGGTTACAAA

[a]All primers are written 5' to 3'.

Table S2. Concentration of human IFN-β in chicken serum.

Chickens	Bird no.	Sex	hIFN-β (ng/ml)
hIFN-β Knock-in	#3619	Female	1.75 ± 0.12
	#3784	Female	0.51± 0.05
	#3607	Female	0.23 ± 0.04
	#3716	Male	n. d.
WT (WL)	#F1	Female	n. d.
	#M1	Male	n. d.

n. d.; not detected, detection limit 10pg/ml

Supplementary Figure 1

IFN-β KI egg white transferred

WT egg white transferred

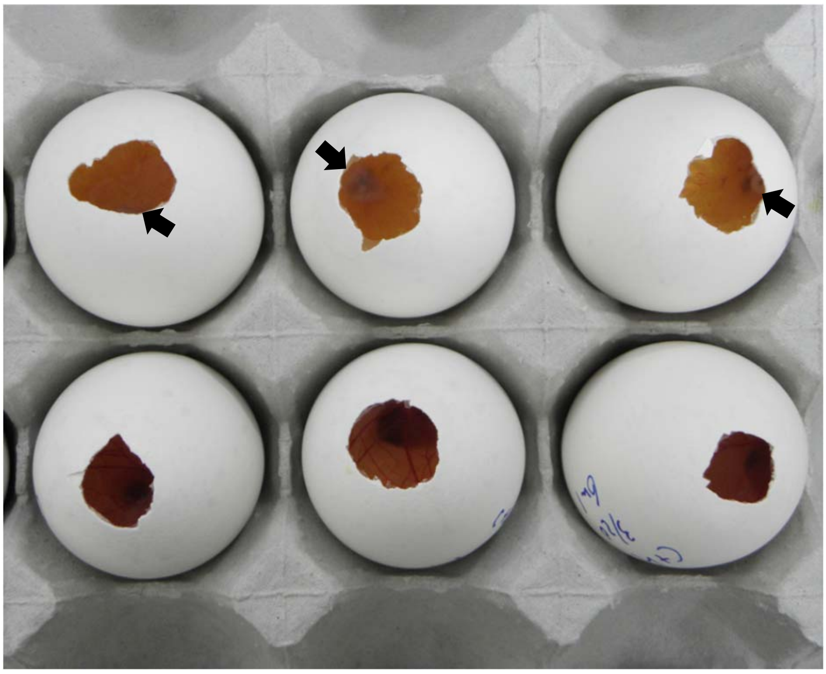

Transplanted egg white	living embryo no.	dead embryo no.	% of dead embryo
hIFN-β	4	17	81.0*
WT	12	9	42.9

*Significantly different compared with WT, as calculated by a chi-square test ($P < 0.05$).

Detrimental effect of IFN-b KI egg white on chicken embryogenesis. Egg white from IFN-b KI eggs and WT was transferred to windowed WT fertilized eggs. The eggs were incubated, and embryo viability was assessed. The image represents typical embryos on day 6, to which was added IFN-b KI egg white (upper row) or WT egg white (lower row). Arrows indicate the dead embryos. The numbers of dead and living embryos were counted on day 7, and the results are shown in the table.

Supplementary Figure 2

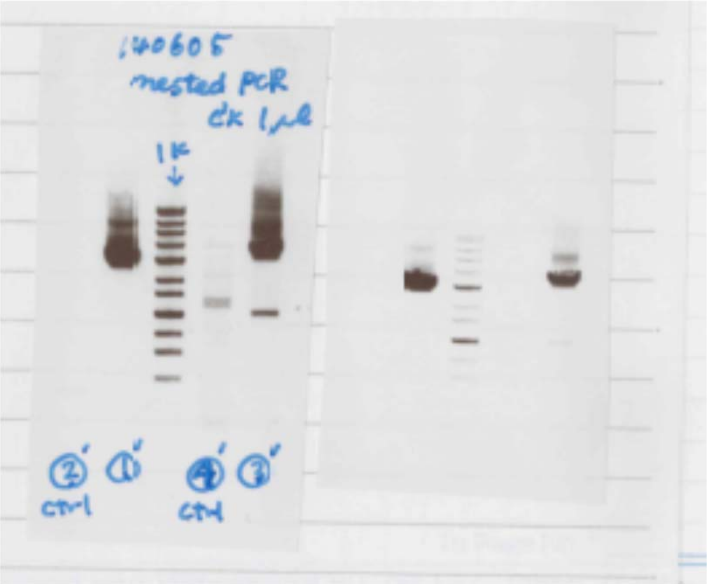

PCR amplification of the donor cassette knocked in at the *OVA* locus in the PGC genome (Fig. 1b).

Supplementary Figure 3

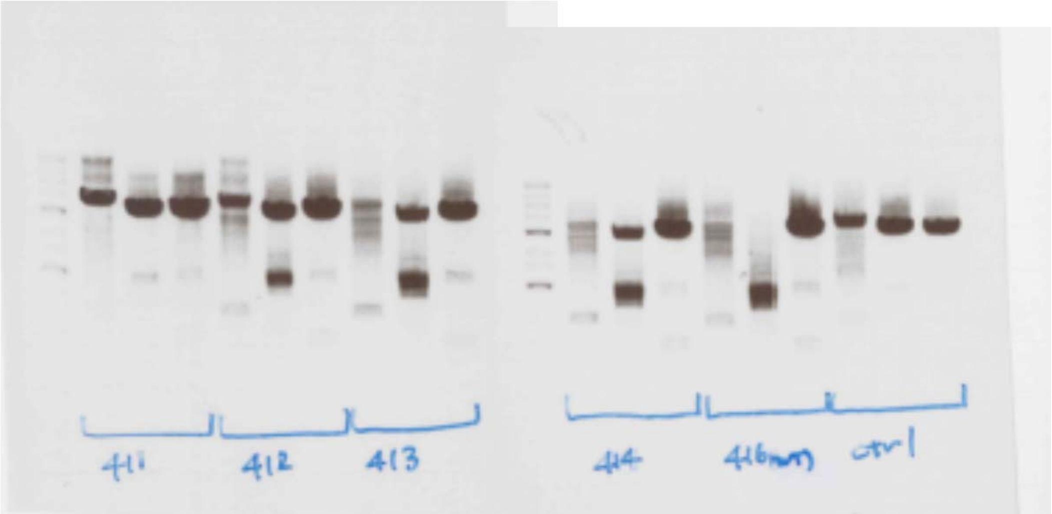

KI *hIFN-β* in semen of chimeric G0 roosters (Fig. 2b).

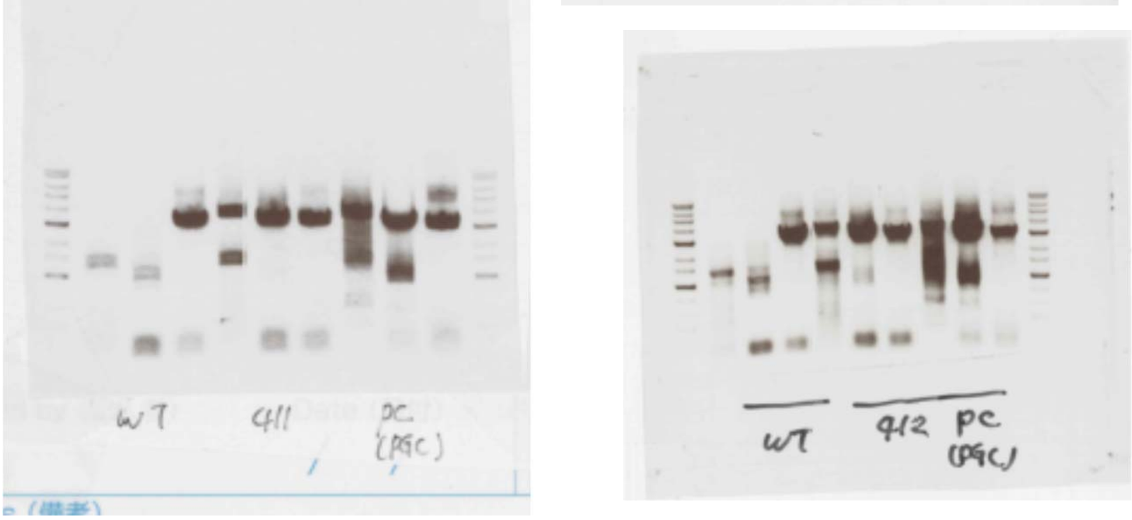

KI *hIFN-β* in the G1 chickens (Fig. 2c).

Supplementary Figure 4

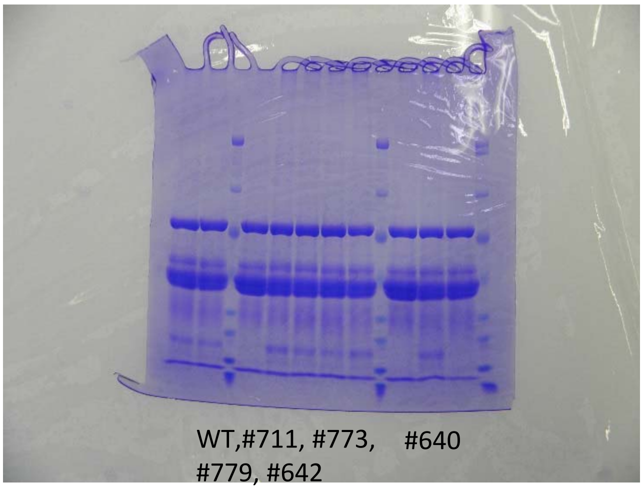

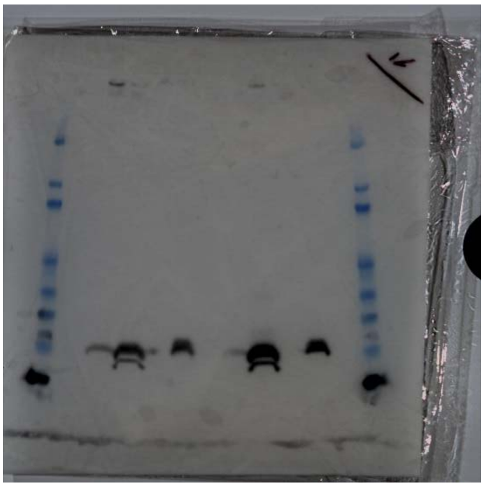

#640
Blot: anti hIFN-β Ab

Production of hIFN-β in the egg white of eggs from KI hens (Fig. 4d-f).
